# Spinning Out of Control: A 19-Year-Old Female with Spinning-Related Exertional Thigh Compartment Syndrome

**DOI:** 10.7759/cureus.939

**Published:** 2016-12-24

**Authors:** Daniel J Gould, Ido Badash, Sukgu Han, Alex K Wong

**Affiliations:** 1 Division of Plastic and Reconstructive Surgery, Keck School of Medicine of USC; 2 Medical School, Keck School of Medicine of USC; 3 Division of Vascular Surgery, Keck School of Medicine of USC

**Keywords:** compartment syndrome, spinning, rhabdomyolysis, thigh, stationary bike, exertional injury

## Abstract

Thigh compartment syndrome (TCS) is a rare condition caused by high pressures within the fascial compartments of the thigh, impeding capillary flow and leading to decreased perfusion, tissue hypoxia, and necrosis. TCS is most frequently associated with trauma and anticoagulation but has also rarely been associated with exercise-related injury. We present the case of a 19-year-old female who reported painful swelling in her thighs and darkening of her urine after participating in a spinning class. On physical examination, the patient was found to have tight, painful thigh compartments with extreme tenderness on passive motion. Labs revealed a marked elevation of creatine kinase and leukocytosis. The patient was diagnosed with TCS and underwent emergent decompression fasciotomy and aggressive IV fluids for protection against myoglobinuria. Due to high clinical suspicion, prompt diagnosis, and early surgery, the patient experienced excellent recovery without functional deficits.

## Introduction

Thigh compartment syndrome (TCS) is a medical emergency that occurs when high pressures within the fascial compartments of the upper leg impede the flow of blood in capillaries. The initial lack of perfusion leads to pain and increased swelling within the closed compartment, and tissue hypoxia and necrosis eventually ensue. Therefore, TCS can be limb-threatening, life-threatening, or cause permanent neuromuscular deficits if left untreated [1].

TCS typically occurs in the context of high energy trauma. TCS caused by bleeding into the fascial compartment, resulting from anticoagulation, underlying coagulopathy, or spontaneous hematoma, has also been described [2]. On rare occasions, TCS may be associated with vigorous exercise, including spinning classes [3-4].

Most cases of exertional TCS are chronic in nature, as the large width of the thigh allows significant swelling to occur before the onset of circulatory damage. By contrast, acute TCS is a much rarer clinical entity. In this case report, we describe a patient who developed acute bilateral TCS following a high-intensity spinning class involving a stationary bike. 

## Case presentation

The patient was a previously healthy 19-year-old Caucasian female college student who presented to the hospital with pain and swelling of the thighs bilaterally. Three nights prior to admission, she had attended a spinning class at a local gym for the first time. She stated that the session was intense and lasted 45 minutes, and although she exercised daily, the intensity of this class was outside of her normal level of activity. After the class, she noted swelling and pain in her thighs but did not seek medical attention until three nights later when she noticed that her urine was dark in color. Alarmed by the changes in her urine, the patient presented to the student health center, where she was noted to have rapid progression of thigh swelling accompanied by increasing pain. As a result of these findings, the patient was immediately transferred to the emergency department for further evaluation.

**Figure 1:**
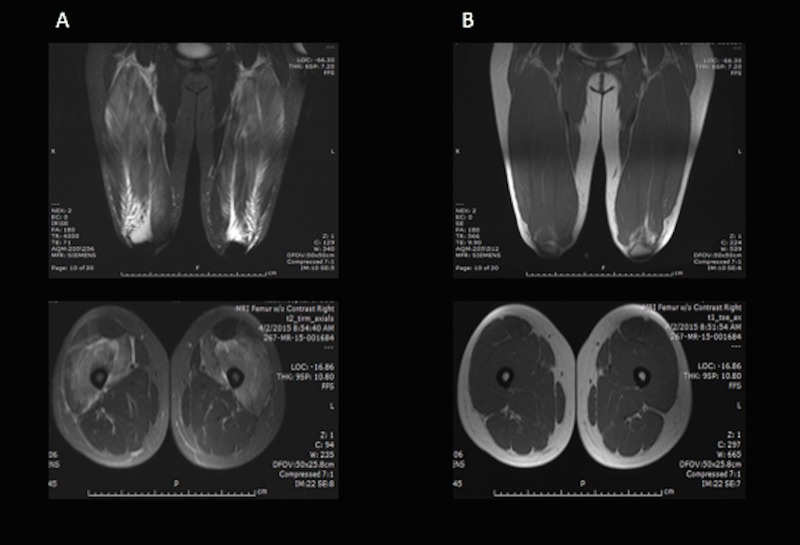
MRI Images of the Thigh Show Swelling in the Anterior Compartment with a Feather-like Signal within the Vastus Musculature Bilaterally A) T2-weighted MRI, B) T1-weighted MRI

Initial assessment revealed marked elevation of creatine kinase (CK) of 53,000 IU/L and leukocytosis of 11,260 white blood cells per microliter. The admitting team ordered an MRI of the lower extremities, which showed swelling in the anterior compartment with a feathery, high-intensity, fluid-sensitive signal within the vastus intermedius, lateralis, and medialis bilaterally (Figure 1).

The pain grew worse overnight, and repeat labs showed a CK of 119,000 IU/L. Vascular surgery was consulted within 12 hours of admission for evaluation of possible compartment syndrome. The patient was found to have tight, painful thigh compartments with extreme tenderness on passive motion. She had normal distal pulses but had developed tingling and numbness in her right leg since admission. The compartment pressures of the right thigh were measured at the bedside and found to be 28 mmHg in the anterior compartment, 14 mmHg in the middle, and 8 mmHg in the posterior. Based on these findings, the patient was diagnosed with TCS. 

Informed patient consent was obtained prior to treatment. No identifying patient information is contained in this report.

**Figure 2:**
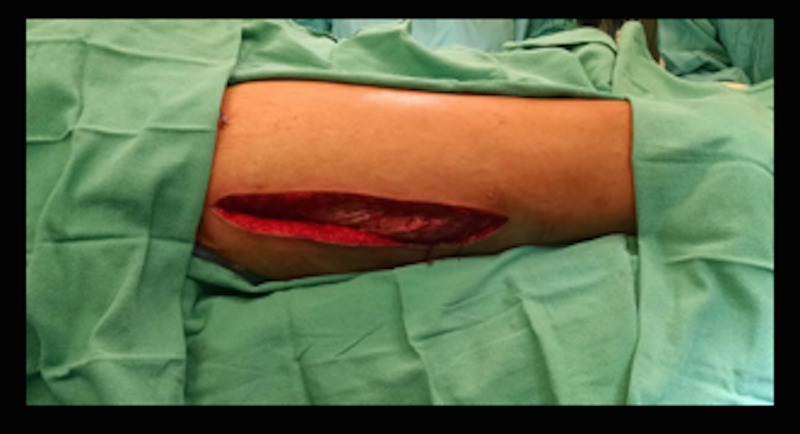
Lateral Incision Used to Access the Anterior, Lateral and Posterior Thigh Compartments. Incision was performed bilaterally.

The patient was then taken to the operating room (OR) for decompression fasciotomy. In the OR, pressures were measured again on both sides, and the patient was found to have anterior compartment pressures of 28 mmHg and 26 mmHg on the right and left sides, respectively. The middle compartment pressures were 15 mmHg on the right, 13 mmHg left, and the posterior compartment pressures were 8 mmHg on the right and 7 mmHg on the left. During the operation, a lateral approach was used to ensure entry and decompression of all three compartments (Figure 2).

**Figure 3:**
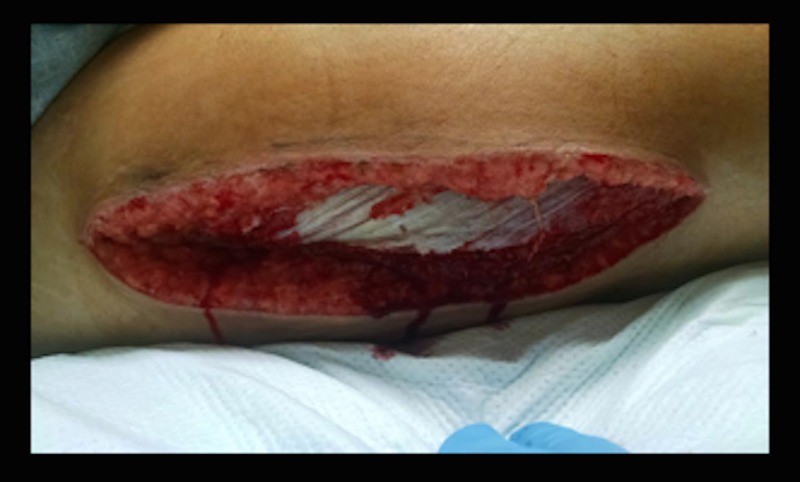
Open Wound at Postoperative Day 3 Shows Proper Healing Ready for Wound Closure

Intraoperative findings included bulging muscle bellies, which appeared initially congested, but remained viable during the case. The muscles reacted to Bovie® electrical stimulation (Bovie Med Corp, Clearwater, FL) bilaterally, and no debridement was required. Wet to dry dressings were placed with a plan for interval wound checks and closure by Plastic Surgery. Postoperatively, her pain improved and her sensation of tingling and numbness resolved. Her repeat CK decreased to 83,000 IU/L, and she was placed on aggressive IV fluids for renal protection against myoglobinuria. Serial metabolic panels were checked while monitoring recovery from rhabdomyolysis. On postoperative day 3, her wounds were appropriate for closure and she was taken by Plastic and Reconstructive Surgery to the OR. Her wound appeared healthy (Figure 3), and she was closed primarily without tension with several drains in place.

Following closure, her recovery was uneventful. The patient recuperated in the rehabilitation facility and was discharged from the hospital approximately two weeks after admission.

## Discussion

In our review of the literature, we found two additional cases that reported TCS of the anterior compartment following spinning [3-4]. Other studies have reported exertional TCS resulting from various exercises, including squats [5], running [6], horse riding [7], and basketball [8]. Although there have only been a few reported cases of exercise-induced TCS in the literature, clinicians should begin to more frequently consider TCS in the context of exertional injuries as high-intensity exercises like spinning become increasingly common.

It is not entirely clear why our patient developed TCS among the millions of other individuals who attend spinning classes. The patient was a college student and stated that she was a social drinker, which makes it possible that acute alcohol use in the days surrounding her spinning class contributed to the development of TCS [9]. Additionally, this was her first spinning class, and she stated that the intensity of the exercise was outside of her normal level of activity. Irregular high-intensity exercise, combined with potential stressors, such as alcohol use, may have led to rhabdomyolysis, edema, and increased compartment pressure that resulted in TCS. Other possible genetic and environmental factors, such as adenosine triphosphate deficiency, hyper-/hypothyroidism, or malnutrition, may play a role in predisposing certain patients to exertional TCS [9].

Although an MRI was ordered by the admitting team in this case due to the ambiguity of the patient’s symptoms and rarity of acute exertional TCS, it is important to stress that TCS is a clinical diagnosis and imaging is not usually needed. In fact, unnecessary imaging can delay diagnosis and surgery, thus increasing the risk of preventable complications. Clinical findings of pain on passive motion, increasing pain at rest and tenderness, paresthesia, pallor, paralysis, pulselessness, poikilothermia, and new onset neurovascular signs all suggest the presence of compartment syndrome. The diagnosis can be confirmed by measuring compartment pressures, with pressures around 30 to 45 mmHg typically indicating TCS and necessitating surgical intervention [5]. 

A distinguishing feature of TCS that makes accurate diagnosis difficult is the delayed presentation of symptoms. This delay may be explained by the fact that the thighs have a relatively large potential space within the compartments, allowing increased swelling to occur before reaching the threshold for developing compartment syndrome. These patients may present with a slow clinical course, and the baseline bulk of thigh musculature further makes diagnosing TCS difficult. Therefore, physicians should maintain a high clinical suspicion for TCS as a missed diagnosis can result in catastrophic permanent functional deficits and complications that include ischemic contracture, infection, and crush syndrome [10]. Accurate clinical diagnosis followed by emergent decompression are imperative to preventing these complications.

Following prompt diagnosis and fasciotomy, recovery from thigh syndrome can be excellent. Our patient was diagnosed early based on clinical suspicion and underwent surgery within 24 hours of admission, resulting in recovery without complications and a return to normal activities following rehabilitation. This demonstrates that a high clinical suspicion for TCS and rapid surgery can prevent permanent functional deficits from TCS.

## Conclusions

We conclude that our patient developed TCS and rhabdomyolysis following a high-intensity spinning class. As these and other high-intensity exercises become more prevalent, clinicians should consider exercise-induced TCS when evaluating patients for pain and neurovascular symptoms in the thigh. Early diagnosis and surgical decompression are critical to prevent the development of irreversible muscle damage and associated functional deficits. However, diagnosis may be difficult due to the subtle and delayed clinical symptoms of TCS. Detailed knowledge of the clinical signs of TCS, a high index of suspicion in patients with thigh injuries, and effective decompression technique can result in complete recovery and good functional outcomes. 
